# Transforming Health Care Through Chatbots for Medical History-Taking and Future Directions: Comprehensive Systematic Review

**DOI:** 10.2196/56628

**Published:** 2024-08-29

**Authors:** Michael Hindelang, Sebastian Sitaru, Alexander Zink

**Affiliations:** 1 Department of Dermatology and Allergy TUM School of Medicine and Health Technical University of Munich Munich Germany; 2 Pettenkofer School of Public Health Munich Germany; 3 Institute for Medical Information Processing, Biometry and Epidemiology (IBE) Faculty of Medicine Ludwig-Maximilian University, LMU Munich Germany; 4 Division of Dermatology and Venereology, Department of Medicine Solna, Karolinska Institute Stockholm Sweden

**Keywords:** medical history-taking, chatbots, artificial intelligence, natural language processing, health care data collection, patient engagement, clinical decision-making, usability, acceptability, systematic review, diagnostic accuracy, patient-doctor communication, cybersecurity, machine learning, conversational agents, health informatics

## Abstract

**Background:**

The integration of artificial intelligence and chatbot technology in health care has attracted significant attention due to its potential to improve patient care and streamline history-taking. As artificial intelligence–driven conversational agents, chatbots offer the opportunity to revolutionize history-taking, necessitating a comprehensive examination of their impact on medical practice.

**Objective:**

This systematic review aims to assess the role, effectiveness, usability, and patient acceptance of chatbots in medical history–taking. It also examines potential challenges and future opportunities for integration into clinical practice.

**Methods:**

A systematic search included PubMed, Embase, MEDLINE (via Ovid), CENTRAL, Scopus, and Open Science and covered studies through July 2024. The inclusion and exclusion criteria for the studies reviewed were based on the PICOS (participants, interventions, comparators, outcomes, and study design) framework. The population included individuals using health care chatbots for medical history–taking. Interventions focused on chatbots designed to facilitate medical history–taking. The outcomes of interest were the feasibility, acceptance, and usability of chatbot-based medical history–taking. Studies not reporting on these outcomes were excluded. All study designs except conference papers were eligible for inclusion. Only English-language studies were considered. There were no specific restrictions on study duration. Key search terms included “chatbot*,” “conversational agent*,” “virtual assistant,” “artificial intelligence chatbot,” “medical history,” and “history-taking.” The quality of observational studies was classified using the STROBE (Strengthening the Reporting of Observational Studies in Epidemiology) criteria (eg, sample size, design, data collection, and follow-up). The RoB 2 (Risk of Bias) tool assessed areas and the levels of bias in randomized controlled trials (RCTs).

**Results:**

The review included 15 observational studies and 3 RCTs and synthesized evidence from different medical fields and populations. Chatbots systematically collect information through targeted queries and data retrieval, improving patient engagement and satisfaction. The results show that chatbots have great potential for history-taking and that the efficiency and accessibility of the health care system can be improved by 24/7 automated data collection. Bias assessments revealed that of the 15 observational studies, 5 (33%) studies were of high quality, 5 (33%) studies were of moderate quality, and 5 (33%) studies were of low quality. Of the RCTs, 2 had a low risk of bias, while 1 had a high risk.

**Conclusions:**

This systematic review provides critical insights into the potential benefits and challenges of using chatbots for medical history–taking. The included studies showed that chatbots can increase patient engagement, streamline data collection, and improve health care decision-making. For effective integration into clinical practice, it is crucial to design user-friendly interfaces, ensure robust data security, and maintain empathetic patient-physician interactions. Future research should focus on refining chatbot algorithms, improving their emotional intelligence, and extending their application to different health care settings to realize their full potential in modern medicine.

**Trial Registration:**

PROSPERO CRD42023410312; www.crd.york.ac.uk/prospero

## Introduction

Taking a patient’s medical history is of central importance in the health care sector. Collecting comprehensive data is essential for accurate diagnosis and customized treatment [1]. Traditionally, clinicians have relied on interviews or questionnaires to gather this important information, but these methods can lack efficiency and accuracy, potentially leading to incomplete records and low patient engagement [2]. New technologies have brought about innovative solutions to streamline documentation, such as chatbots, with their ability to digitally transform data collection [3]. Chatbots can use artificial intelligence (AI) and natural language processing (NLP) to simulate conversations and minimize the limitations of paper-based processes [4-6]. The integration of chatbots promises significant improvements in care by enabling accurate, streamlined documentation that supports personalized, evidence-based clinical decision-making and greater patient engagement [7,8]. While chatbots are widely used in other areas, such as entertainment, customer service [9], security systems, and emergency communications [10-12], there is a lack of thorough research evaluating their effectiveness, usability, and acceptability of chatbots specifically for health care data collection. Research has focused on a narrow area without contextualizing the broader implications. To date, few people have had access to sophisticated AI due to its cost and complexity. However, new publicly available models, such as ChatGPT, are making these capabilities accessible to a wide audience by analyzing large amounts of literature and data in seconds to make time-critical decisions in a more data-driven and accurate way [13-17]. For interactions in the health care sector, specific and individual patient profiles can be addressed in order to improve documentation and the associated health outcomes. In addition, continued adoption will ensure that counseling by health care professionals remains widely accessible, especially in underserved communities [18]. In addition, their ability to work continuously and remotely can improve health care by ensuring that expert-level advice is always available, improving access to quality care, especially in underserved areas [18,19]. However, these benefits must be balanced by robust measures to ensure that the use of AI in health care improves, rather than undermines, patient care and trust [20].

Despite the promise of chatbots, important considerations are taken into account, particularly in health care. Cybersecurity is paramount, as chatbots handle sensitive medical information that must be protected from unauthorized access or data breaches [21,22]. Furthermore, despite the remarkable capabilities of chatbots in effectively processing and generating responses through predefined algorithms, they often lack the empathetic understanding and emotional intelligence inherent in human interactions [23]. This limitation can affect relationship-building and patient trust, especially during sensitive medical conversations [20].

**Figure 1:**
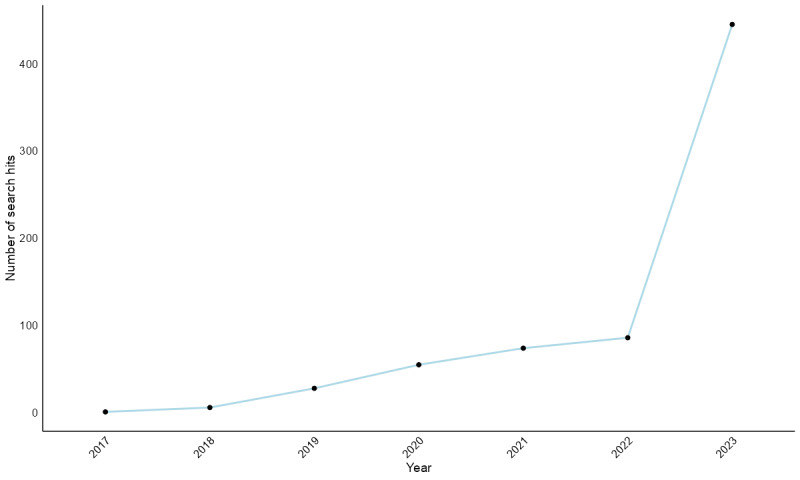
Number of studies over recent years: “chatbot*” AND “medicine.” This chart shows the increasing trend in publications on chatbots in medicine from 2017 to 2023. In 2022, there was an exponential increase in published studies, indicating a growing research interest and progress in chatbots in medicine.

Recent data highlighted the growing interest in the interplay between chatbots and medicine. An analysis of studies from the first study in 2017 to 2024 with the search query “chatbot*” AND “medicine” shows a significant increase, especially in 2022, with the trend rising from a single study in 2017 to 445 in 2023 (Figure 1).

Chatbots rely on advanced algorithms and AI-supported NLP for their technical function. These techniques enable chatbots to examine user input, provide applicable data in the form of feedback, and modify their interactions depending on context and user behavior, which can be refined through machine learning approaches, including information-driven learning and pattern recognition [24-26].

Considering the potential benefits and problems associated with chatbots, a thorough investigation is essential to assess their impact on the process of medical history–taking. While existing studies have examined the practicality and acceptability of chatbots in specific medical areas, such as psychological well-being or genetic counseling, a systematic literature review is needed for a complete understanding of chatbot-based history-taking [27-29].

The primary objective of this systematic review is to provide a comprehensive assessment of the role, effectiveness, usability, and patient acceptance of chatbots in medical history–taking. This systematic review also aims to explore the impact and future directions of integrating chatbots into clinical settings by assessing data accuracy, level of patient interaction, health care provider efficiency, and patient outcomes. Chatbots could transform the process of taking medical histories by supporting the accurate capture of patient information. In addition, this has the potential to increase productivity and improve the quality and delivery of health care services.

## Methods

### Overview

The systematic analysis was conducted in accordance with PRISMA (Preferred Reporting Items for Systematic Reviews and Meta-Analyses) guidelines for reporting systematic reviews to ensure transparency [30]. The protocol was registered under registration number CRD42023410312 in the PROSPERO database of the National Institute for Health Research [31].

### Eligibility Criteria

Eligibility criteria for the studies were based on the PICOS (participants, interventions, comparators, outcomes, study design) framework for assessing participant demographics, types of interventions assessed, study designs, and outcome of interest [32]. We aimed to identify research investigating chatbots to facilitate medical history–taking to support physicians in diagnosis and treatment planning. The scope was limited to chatbots that facilitate patient disclosure of personal health information to improve accuracy and support clinical decision-making. In contrast, chatbots designed exclusively as “symptom-checkers,” such as stand-alone apps providing rapid assessments and potential diagnoses, were excluded. This exclusion was made to focus on tools that facilitate comprehensive medical history–taking rather than immediate symptom-based advice. There were no limitations on the modality of chatbot input and output. The comparators were not subjected to any specific restrictions. The outcomes of interest included the feasibility, acceptability, and efficacy of chatbot-based history-taking interventions. There were no restrictions on study design, except for conference papers, which were excluded to ensure the inclusion of studies with rigorous peer review and substantial data reporting. The review was limited to English-language studies because resources were limited.

### Information Sources

PubMed, CENTRAL, Embase, MEDLINE (through Ovid), Scopus, and Open Science were searched to identify relevant studies. In addition, reference lists of relevant studies were screened manually.

### Search Strategy

For each database, we developed a search strategy that included keywords, subject headings, mesh terms (in PubMed), filters, and restrictions to find relevant studies. The search terms focused on chatbots, anamnesis, history-taking, and related concepts: (“chatbot*” OR “conversational agent*” OR “chatterbot*” OR “virtual assistant” OR “intelligent virtual agent” OR “artificial intelligence chatbot” OR “AI chatbot” OR “conversational AI” OR “dialogue system”) AND (“anamnesis” OR “medical history” OR “history-taking” OR “medical interview” OR “patient interview” OR “medical questionnaire” OR “patient questionnaire”). The last search was done in July 2024 (Multimedia Appendix 1). Additionally, a reference list search was conducted.

### Selection Process

The selection process was done by 2 authors (MH and SS) independently screening the titles and abstracts of the identified studies based on the predetermined eligibility criteria. Potentially relevant studies were retrieved in full text and further assessed for eligibility. The full-text assessment was also performed independently (MH and SS). Any disagreements between the 2 authors were resolved through discussion, focusing on the eligibility criteria and study relevance. If consensus could not be reached, the involvement of a third author (AZ) was sought when necessary.

### Data Collection Process

Data from the selected studies were extracted independently (MH and SS) using a data extraction form based on the PICO criteria (STROBE [Strengthening the Reporting of Observational Studies in Epidemiology]) [32,33]. The extracted data included information such as the first author, number of authors, country, year, title of the scientific journal, topics and type of journal, impact factor, and main results focused on history-taking (anamnesis). Additional data collected encompassed study design, setting, sample size, type of participants, female percentage, mean age (range), and results. Outcomes extracted focused on key aspects such as feasibility, acceptability, and efficacy. When full-text access was unavailable, the corresponding author was contacted by email. Data were visualized using the R-package for creating alluvial diagrams [34]. Any discrepancies in data extraction were resolved through a discussion between the 2 authors (MH and SS).

### Quality Assessment

The methodological quality of the included observational studies was assessed using the STROBE criteria [33]. Each study was evaluated based on the fulfillment of the STROBE criteria. The studies were categorized into 3 categories: category A, if more than 80% of the STROBE criteria were fulfilled; category B, if 50%-80% were met; and category C if less than 50% of the criteria were fulfilled [35]. For example, category A studies provided comprehensive details on study objectives, participant selection, and statistical analysis. Category B had adequate but incomplete information. Category C studies frequently lacked critical details such as clear definitions of eligibility criteria or thorough data collection methods.

In addition, the RCTs included in this review were evaluated for risk of bias using the Risk of Bias tool and the robvis R-package [36,37]. The RoB 2 tool assesses various domains of bias, including randomization, allocation concealment, blinding, incomplete outcome data, selective reporting, and other potential sources of bias. The overall risk of bias score was determined for each study based on the number of criteria for high risk of bias met. Studies are considered to have a low risk of bias if no domains are rated as high risk and most domains are rated as low risk. Studies with some concerns in one or more domains but no high-risk ratings are considered to have some concerns. If any domain is rated as high risk, the study is considered to have a high risk of bias.

### Software and Tools

Data were managed and analyzed using R (version 4.2.1; The R Foundation). The ggplot2 package [38] was used for data visualization and the robvis R-package was used for risk of bias charts [37]. The alluvial R package [34] was used to create alluvial diagrams.

## Results

### Study Selection

**Figure 2:**
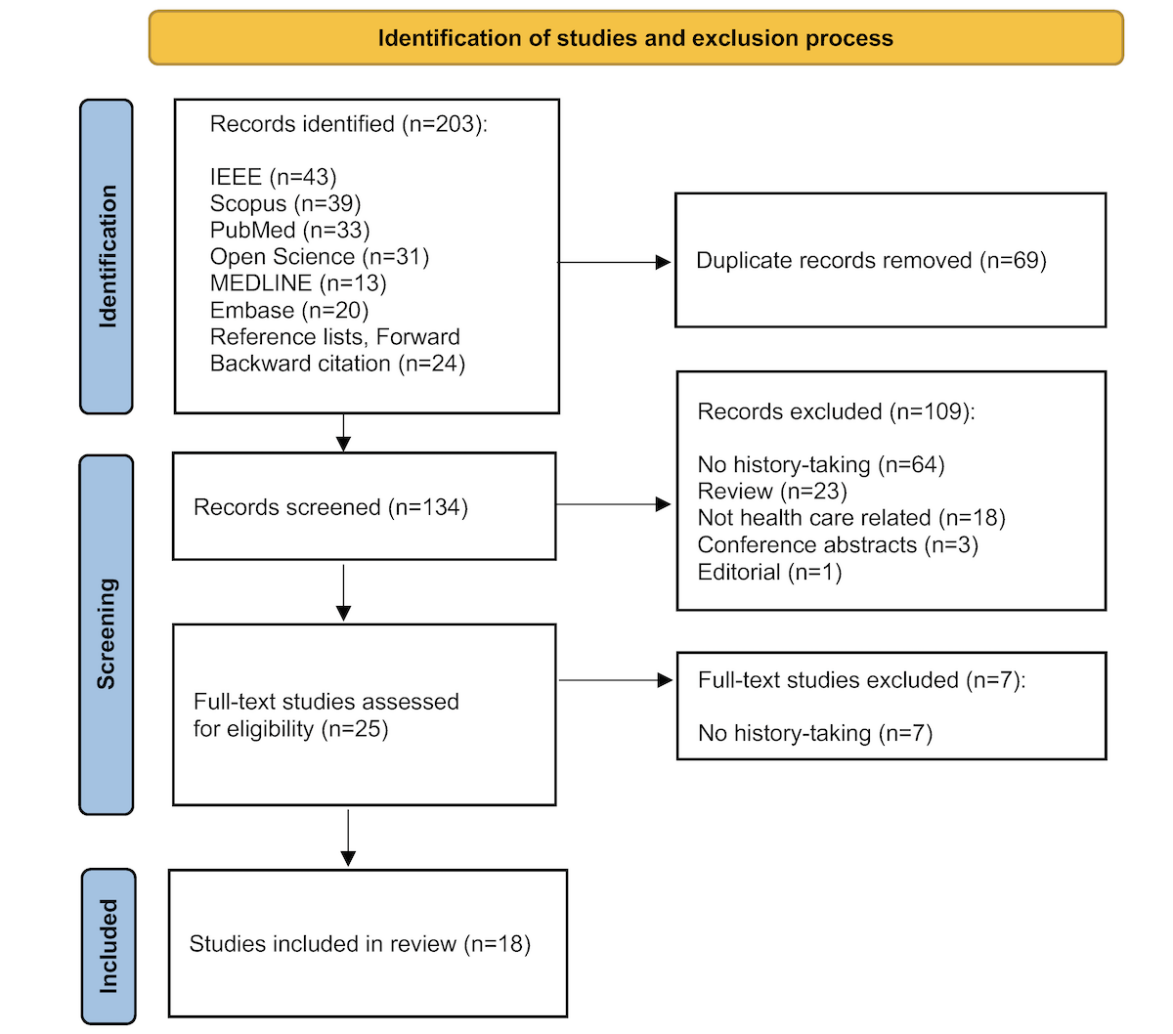
Flowchart of the study search and inclusion. This flowchart details the systematic process of selecting studies for the review, starting from 203 records and narrowing down to 18 studies after removing duplicates and applying eligibility criteria. IEEE: Institute of Electrical and Electronic Engineers.

The initial literature search yielded 203 records. After removing 69 duplicate studies, a total of 134 unique records were screened based on titles and abstracts. Of these, 109 studies did not meet the eligibility criteria and were excluded. Subsequently, 25 full-text studies were screened, resulting in 18 studies being included in the review (Figure 2).

### Study Characteristics

The studies investigated the use of chatbots for history-taking across diverse patient populations and sample sizes (range: n=5-61,070) and were mostly published in scientific health technology journals with varying impact factors (mean 4.52, SD 4.49; range: 0.14-14.71; Table 1). The studies used different research designs, including 9 cross-sectional studies, 3 case-control studies, 2 observational studies, and 3 RCTs (Multimedia Appendix 1 and Tables 1-3).

**Table 1:** General characteristics of the included studies. This table summarizes the number of authors, countries, and journal topics of the studies, showing most research from Germany and the United States, and a focus on Health Informatics and Technology.

			Count, n (%)
**Numbers of authors**			
	1-3		4 (22)
	4-6		8 (44)
	>6		6 (33)
**Countries**			
	Germany		6 (33)
	United States		6 (33)
	Switzerland		3 (17)
	Australia		2 (11)
	New Zealand		1 (6)
**Scientific journals**			
	**Topics of scientific journals**		
		Health Informatics and Technology	12 (67)
		Medical Imaging and Radiology	2 (11)
		Genetics and Genetic Counseling	2 (11)
		Surgical Procedures and Techniques	1 (6)
		Mental Health and Psychology	1 (6)

**Table 2:** Study characteristics. This table details study characteristics, including author, year, design, sample size, participant type, and key findings, highlighting diverse participant demographics and study outcomes.

Reference			Participants					Methods and result	
Authors (year)	Study design	n		Type of participants	Female (%)	Mean age (years)	Type of measurement		Relevant results
Denecke et al (2018) [39]	Cross-sectional study	22		Music therapy patients	41	39 (range 19-73)	Usability test of the tool and corresponding questionnaire		CUI^a^-based self-anamnesis app well-received, potential for collecting anamnesis data.
Denecke et al (2022) [40]	Cross-sectional study	5		Radiology patients	40	39.2 (range 17-73)	System usability scale		Digital medical interview assistant with good usability.
Faqar-Uz-Zaman et al (2022) [41]	RCT^b^	450		Patients with abdominal pain in ER^c^	52.2	44 (range 18-97)	Accuracy of diagnosis by ER doctor and Ada app according to the final diagnosis		Classic patient-physician interaction superior to AI^d^-based tool, but AI benefits diagnostic efficacy.
Frick et al (2021) [42]	Cross-sectional study	148		German participants	53	33.32 (SD 12.59)	Scales for disclosure and concealment of medical information		Patients prefer disclosing to physicians over chatbots. No significant difference in concealment.
Gashi et al (2021) [43]	Cross-sectional study	N/A^e^		N/A	N/A	N/A	N/A		AnCha chatbot improves patient-doctor communication, enhances diagnostic process.
Ghosh et al (2018) [44]	Case-control study	30 scenarios		Not specified	N/A	N/A	True positives and false positives, precision		Medical chatbot helps with automated patient preassessment.
Heald et al (2021) [27]	Feasibility study	506		Various types of care	58	56.6 (SD 12.5)	Colon cancer risk assessment tool		Chatbot feasible for increasing genetic screening in at-risk individuals.
Hennemann et al (2022) [45]	Observational study	49		Adult patients from an outpatient psychotherapy clinic	61	33.41 (SD 12.79)	Interviews, questionnaires, diagnostic software		Chatbot shows moderate to good accuracy for condition suggestions.
Hong et al (2022) [46]	Cross-sectional study	20		Primary care patients	60	50	Web-based survey		Patients believe chatbot helps clinicians better understand their health.
Ireland et al (2021) [28]	Cross-sectional study	83		Adults who had whole exome sequencing for genetic condition diagnosis	53	range 23.2-80.4	Transcript analysis		Chatbot enhances genetic counseling by providing genomic information.
Jungmann et al (2019) [47]	Case-control study	6		Psychotherapists, psychology students, and laypersons	50	40 (therapists) 22 (students)	Case vignettes, health app comparison		Chatbot shows moderate diagnostic agreement, improvement needed for childhood disorders.
Nazareth et al (2021) [48]	Retrospective, observational study	61,070		Women’s health	96	N/A	Genetic testing results		Chatbot helps identify patients at high risk for hereditary cancer syndromes.
Ni et al (2017) [49]	Cross-sectional study or proof-of-concept	11		Patients with chest pain, respiratory infections, headaches, and dizziness	N/A	N/A	Question accuracy, prediction accuracy		Chatbot generates medical reports with varying accuracy based on disease category.
Ponathil et al (2020) [50]	Cross-sectional study	50		Adults	50	N/A	NASA Task Load Index workload instrument IBM Usability Questionnaire Technology Acceptance Model Questionnaire		Chatbot interface saves time, preferred for collecting family health history.
Reis et al (2020) [51]	Case-control study	16		Physicians	35	35.51	N/A		Failure of cognitive agent highlights need for managing resistance and transparency.
Schneider et al (2023) [52]	RCT	30		Hymenoptera venom allergic patients	N/A	38.93 (SD 12.56)	Standardized questionnaire		Chatbot-supported anamnesis saves time, potential for allergology assessments.
Wang et al (2015) [29]	RCT, hospital	70		Majority of patients from underserved populations (low-income families, elders, people with disabilities, and immigrants)	60	Majority in age group 45-54	Interview, questions		Technological support for documenting family history risks is accepted and feasible.
Welch et al (2020) [53]	Cross-sectional study	3204		General population	100	49.4 (SD 7.1)	Standardized questionnaire		Chatbot engages users, potential for gathering family health history at population level.

^a^CUI: conversational user interface.

^b^RCT: randomized controlled trial.

^c^ER: emergency room.

^d^AI: artificial intelligence.

^e^Not applicable.

**Table 3:** Chatbot characteristics. This table outlines the chatbots used in the studies, including their name, goal, modality, techniques, outcomes, user preferences, and challenges, showcasing varied applications and technological approaches in health care. Table format based on Schachner et al [54].

Authors (year)	Name	Goal	Modality	Techniques	Main outcomes	User preference	Challenges
Denecke et al (2018) [39]	Ana	Collect medical history for music therapy	Mobile app: Text input	AIML^a^, rule-based	Comprehensive data collection, usability	Engaging, intuitive	Integration, diverse interactions, data completeness
Denecke et al (2022) [40]	Not specified	Improve radiological diagnostics	Telegram CUI^b^	RiveScript (rule-based)	Enhanced knowledgeability, diagnostic quality	User-friendly	Clinical workflow integration, data security
Faqar-Uz-Zaman et al (2022) [41]	Ada	Evaluate diagnostic accuracy in ER^c^	iPad app	AI^d^ questionnaire, ML^e^	Increased diagnostic accuracy	Not specified	Physician integration, diagnostic variability
Frick et al (2021) [42]	Not specified	Elicit truthful medical disclosure	Digital survey	Common CA^f^ technologies	Disclosure versus concealment	Prefer physicians	Information accuracy, privacy
Gashi et al (2021) [43]	AnCha	Collect previsit medical history	IBM Watson, web-based	Rule-based tree	Efficient data collection	Reduces previsit anxiety	Clinical integration, data security
Ghosh et al (2018) [44]	Quro	User symptom check, personalized assessments	Web interface	NLP^g^, ML	Precision in condition prediction	High engagement	Data complexity, accurate predictions
Heald et al (2021) [27]	Not specified	Screen for heritable cancer syndromes	Web-based, text-based	AI conversation, NLP	Efficient risk assessment, facilitated testing	High engagement, completion rates	Workflow integration, genetic risk understanding
Hennemann et al (2022) [45]	Ada	Diagnose mental disorders	App-based symptom checker	AI analysis, NLP	Moderate diagnostic accuracy	Mixed preferences	Diagnostic performance, user input dependency
Hong et al (2022) [46]	Genie	Collect detailed medical histories	Web-based, AI speech-to-text	AI, NLP	Improved history collection	Helpful for PCPs^h^	Ease of use, AI use concerns
Ireland et al (2021) [28]	Edna	Support genomic findings decision-making	Mobile, tablet, PC	NLP, Sentiment Analysis	Enhanced patient agency, informed decisions	Ease of access, supports consent	Empathy, complex interactions, data privacy
Jungmann et al (2019) [47]	Ada	Diagnose mental disorders	Mobile app	AI symptom analysis	Moderate diagnostic agreement	Not specified	Accuracy for complex cases
Nazareth et al (2021) [48]	Gia	Hereditary cancer risk triage	Web-based, mobile	NLP	Automated risk triage, educational interactions	High engagement	Workflow integration, privacy, diverse needs
Ni et al (2017) [49]	Mandy	Automate patient intake	Mobile app	NLP, data-driven analysis	Reduced staff workload, privacy maintenance	Improves physician efficiency	Full clinical integration, privacy, diverse interactions
Ponathil et al (2020) [50]	VCA	Collect family health history	Web-based chat	Not specified	Higher satisfaction, lower workload	Preferred by most users	Multiple clicks, extensive interaction
Reis et al (2020) [51]	Cognitive Agent	Automate anamnesis-diagnosis-treatment	Voice-based AI chatbot	ML, NLP, speech recognition	Reduced documentation time	Reduces nonbillable activities	Physician resistance, legal concerns, oversimplification
Schneider et al (2023) [52]	Not specified	Standardize allergy history-taking	HTML-based, digital	HTML, Java scripting	Time-efficient, accurate history-taking	High satisfaction	Question clarity, specificity
Wang et al (2015) [29]	VICKY	Collect family health histories	Touch-screen tablet	Speech recognition, decision trees	High satisfaction, effective identification	Easy to use, recommended	Data entry issues, complex questions
Welch et al (2020) [53]	It Runs In My Family	Assess hereditary cancer risk	Web-based chatbot	NLP	High engagement, thorough assessments	Prefer chatbot to web forms	Data accuracy, interface design, demographic reach

^a^AIML: artificial intelligence markup language.

^b^CUI: conversational user interface.

^c^ER: emergency room.

^d^AI: artificial intelligence.

^e^ML: machine learning.

^f^CA: conversational agent.

^g^NLP: natural language processing.

^h^PCP: primary care physician.

**Figure 3:**
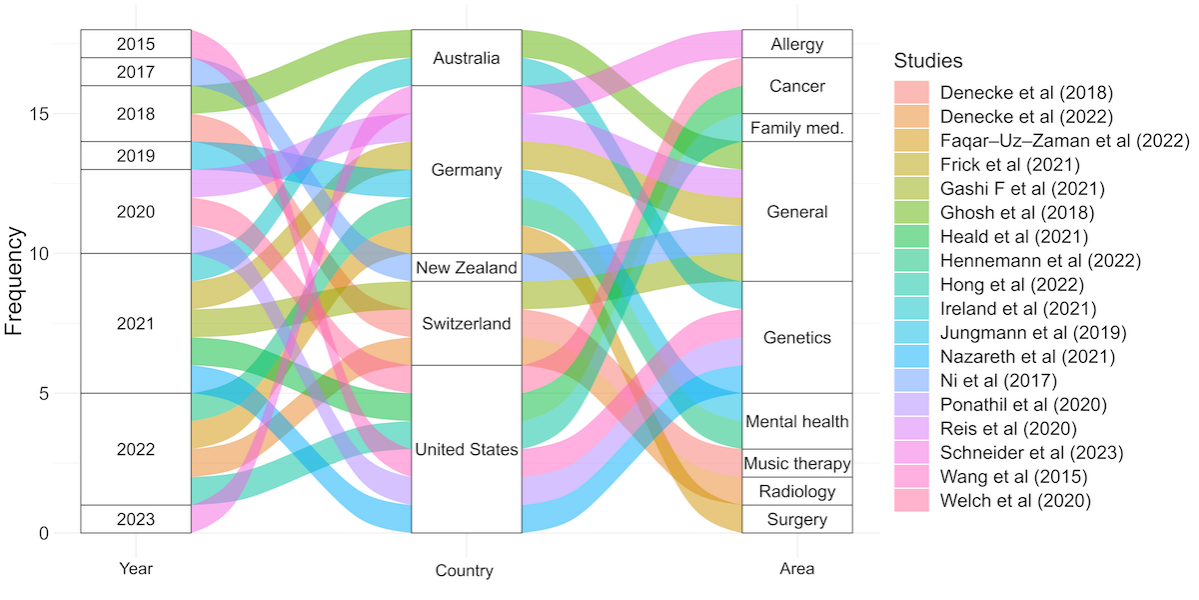
Alluvial diagram of the publication date, country, and area of studies. The alluvial diagram illustrates the distribution of studies by year, country, and medical area from 2015 to 2023, highlighting increased publications in 2020 and 2022, with contributions from Germany, the United States, and Switzerland across various medical fields.

The alluvial diagram (Figure 3 [27,29,39-53]) shows an overview of the literature over time, indicating the year, the country of origin, and the medical area of focus for each study. The included studies were published from 2015 to 2023. Most of the studies were published in 2020 and 2022. The included studies (Figures 3 [27,29,39-53] and 4) were conducted in Switzerland [39,40,43], Germany [41,42,45,47,51,52], the United States [27,29,46,48,50,53], Australia [28,44], and New Zealand [49]. The studies cover a diverse range of medical areas: general medicine [42-44,49,51] genetics [28,29,48,50] cancer research [27,53], family medicine [46], mental health [45,47], radiology [40], surgery [41], allergy [52], and music therapy [39].

**Figure 4:**
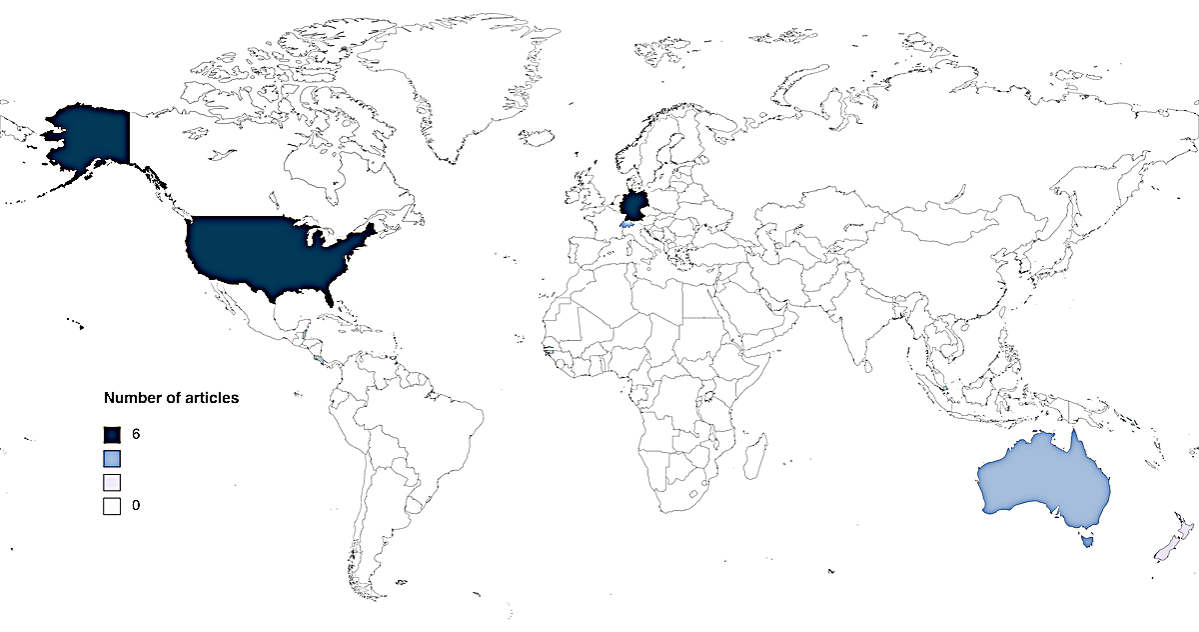
World map showing the number of studies published in each country. This map shows the geographical distribution of the studies, with most research originating from Germany and the United States. Created with MapChart [55].

### Quality Appraisal of the Included Studies

**Figure 5:**
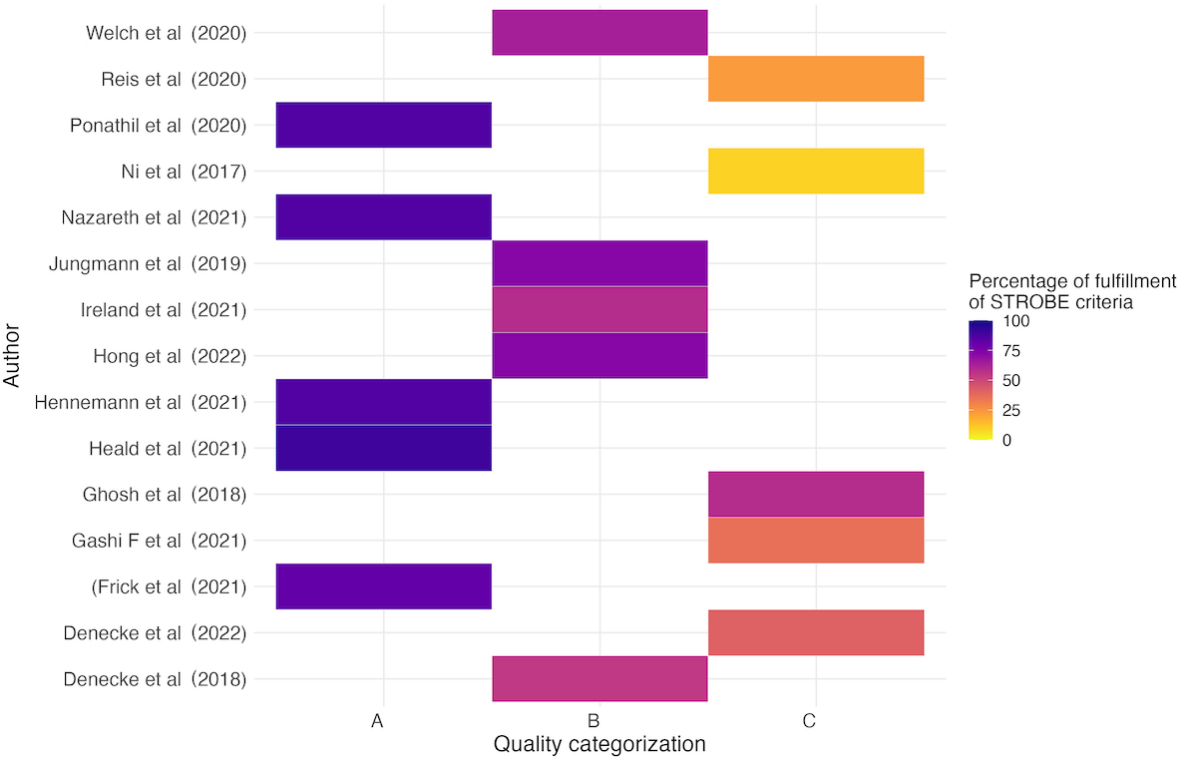
Fulfillment of STROBE criteria and categorization. This bar chart categorizes observational studies by their adherence to STROBE criteria, showing 37.5% of high-quality (category A), and an even split between moderate (category B) and lower quality (category C). STROBE: Strengthening the Reporting of Observational Studies in Epidemiology.

Among the 16 observational studies, 6 (38%) studies were classified as category A [27,42,45,48,50], indicating high methodological quality with more than 80% of the STROBE criteria fulfilled (Multimedia Appendix 1). A total of 5 (31%) studies were classified as category B [28,39,46,47,53], meeting 50%-80% of the STROBE criteria, and 5 (31%) studies were classified as category C [40,43,44,49,51], meeting less than 50% of the STROBE criteria (Figure 5 [27,28,39,40,42-51,53]). The lack of adherence to STROBE criteria in observational studies can have a significant impact on the quality. Missing elements, such as clear definitions of eligibility criteria or participants or detailed methods, lead to biases that reduce validity and reliability. For example, the study of Denecke et al [40] showed a high risk of selection bias due to a small, nonrepresentative sample and lack of eligibility criteria, limiting the generalizability of their findings. Gashi et al [43] faced biases from the absence of a control group and unclear eligibility criteria. This could impact the validity of the effectiveness results. Ghosh et al [44] showed high bias from simulated scenarios without real patient interactions. This could lead to overestimated accuracy and applicability in real-world settings.

**Figure 6:**
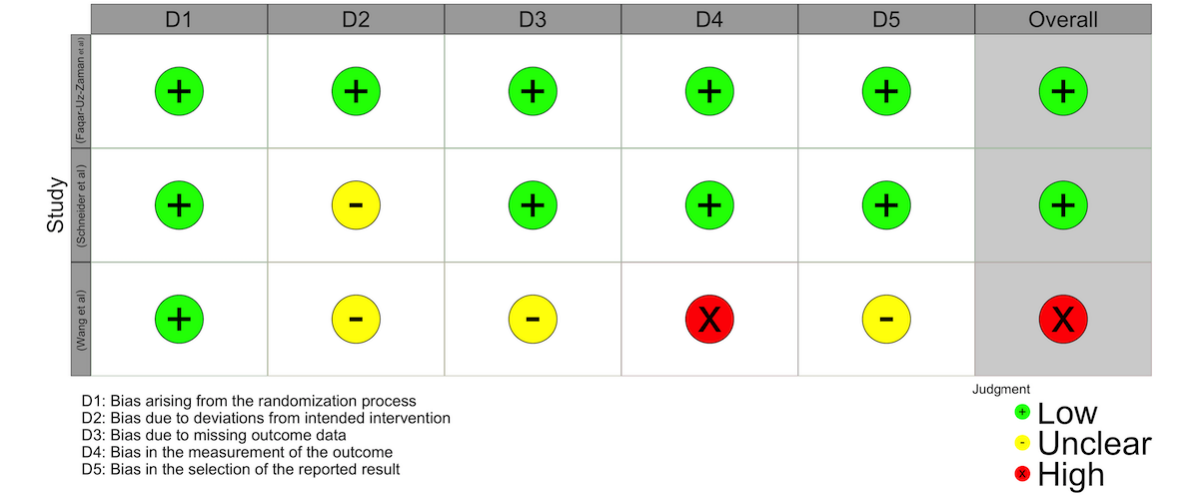
Risk of bias domains (RoB-tool) for randomized controlled trials.

The studies by Schneider et al [52] and Faqar-Uz-Zaman et al [41] showed a low risk of bias according to the RoB tool, with detailed methodology and statistical analysis. In contrast, the study by Wang et al [29] showed a risk of bias due to the absence of intention-to-treat analysis and participants being aware of the intervention (Multimedia Appendix 1 and Figure 6), which could skew results by excluding noncompleters and altering participant behavior.

### Summary of Statistical Analyses

The studies included in this systematic review used a variety of statistical methods. Descriptive statistics summarized demographics and usability ratings. Comparative analyses used 2-tailed *t* tests and chi-square tests to compare diagnostic accuracy and user engagement. κ statistics measured agreement between chatbot and expert diagnoses. Precision and accuracy metrics were assessed using precision, recall, and *F*_1_-scores. Nonparametric tests, such as the Mann-Whitney *U* test showed significant reductions in anamnesis duration. CIs and *P* values were reported where relevant to clarify the strength of the evidence.

### Usability and User Experience of Chatbots

Five studies focused on the usability and user experience of chatbots in history-taking (Tables 2 and 3). Denecke et al [39,40] found that chatbots were well-received by participants and showed potential for history-taking. Usability scores were high, between 90 and 100 (average 96). Ponathil et al [50] found that using a voice-controlled assistant interface for taking family health history significantly reduced history-taking duration. Ghosh et al [44] implemented a medical chatbot that assists with automated patient preassessment through symptom analysis, demonstrating the possibility of avoiding form-based data entry. The chatbot correctly identified at least one of the top three conditions in 83% (n=25) of cases and two out of three conditions in 67% (n=20) of cases. Welch et al [53] found high engagement and interest in chatbots, suggesting the potential for gathering family health history information at the population level in the United States. Of the over 14,000 who participated in the assessment of the study, 54.4% (n=7616) of users went beyond the consent step, and 22.7% (n=3178) of users completed the full assessment.

### Chatbots and Patient-Doctor Communication

One study highlighted the potential of chatbots to improve patient-doctor communication. Gashi et al [43] reported that using a chatbot could reduce patient nervousness, allow patients to respond more thoughtfully, and give physicians a more comprehensive picture of the patient’s condition.

### Diagnostic Accuracy and Efficacy of Chatbots

Nazareth et al [48] found that a chatbot can help identify high-risk patients for hereditary cancer syndromes. A total of 27.2% (n=14,850) of the chatbot users met the criteria for genetic testing, and 5.6% (n=73) of the chatbot users had a pathogenic variant. Ni et al [49] reported that Mandy, a chatbot, automates history-taking, understands symptoms expressed in natural language, and generates comprehensive reports for further medical investigations, with varying degrees of accuracy depending on the disease category. Hennemann et al [45] reported that the app-based symptom checker with an AI chatbot showed agreement with therapist diagnoses in 51% (n=25) of cases for the first condition suggestion and in 69% (n=34) of cases for the top five condition suggestions. Jungmann et al [47] tested a health app’s diagnostic agreement with case vignettes for mental disorders, pointing to the need for improvement in diagnostic accuracy, especially for mental disorders in childhood and adolescence.

### Patient Perceptions and Acceptance of Chatbots

Hong et al [46] reported that most primary care patients believed that chatbots could help clinicians better understand their health and identify health risks. Ireland et al [28] found that the development of the Edna tool, an AI-based chatbot that interacts with patients via speech-to-text, signifies progress toward creating digital health processes that are accessible, acceptable, and well-supported, enabling patients to make informed decisions about additional findings. Heald et al [27] highlighted the feasibility of using chatbots for increasing genetic screening and testing in individuals at risk of hereditary colorectal cancer syndromes.

### Challenges and Limitations of Chatbots

Reis et al [51] noted the importance of managing user resistance and fostering realistic expectations when implementing AI-based history-taking tools. Frick et al [42] found that patients preferred to disclose medical information to a physician rather than a conversational agent.

### Effectiveness on Chatbots

Faqar-Uz-Zaman et al [41] found that classic patient-physician interaction was superior to an AI-based diagnostic tool applied by patients. However, they also noted that AI tools can benefit clinicians’ diagnostic efficacy and improve the quality of care. Schneider et al [52] found that a chatbot-supported anamnesis could save significant time by 57.3%, in assessing Hymenoptera venom allergies with high completeness (73.3%) and patient satisfaction (75%). Wang et al [29] demonstrated that technological support for documenting family history risks can be highly accepted, feasible, and effective.

## Discussion

### Principal Results

This systematic review highlights that the use of chatbots can improve medical history–taking. Results of the included studies have shown that chatbots can facilitate data collection while increasing patient engagement and satisfaction [39,49]. Chatbots show value, especially in collecting structured data such as family history [29,50,53]. As highlighted, the collection of family history benefits significantly from chatbot automation due to the simple nature of their queries, which typically require binary responses. This area contrasts with the challenges of collecting data on undiagnosed symptoms, where patient responses are inherently more nuanced and variable. The inherent abilities of chatbots to handle yes or no questions efficiently and without misinterpretation make them particularly valuable in this context, minimizing human error and optimizing the data collection process. Several studies have highlighted that chatbots provide a more engaging patient interaction, often perceived as less intimidating than traditional face-to-face conversations [27,46]. This interaction is crucial as it motivates patients to disclose more comprehensive health information, which can lead to better health outcomes. While chatbots excel at retrieving and conveying information through interactions that require limited context, their capabilities remain limited when it comes to more nuanced understanding and complex emotions. Research has shown that specific sensitive topics are best-discussed face-to-face with a human, where building trust is paramount [42]. Chatbots, on the other hand, offer relief through constant availability and allow patients to share details from any location and at any time, which can expand access—especially for urgent needs that require quick access to medical history [41,53]. This expanded access aims to improve care, especially in cases where timely data can make the difference between outcomes. In addition, chatbots support overburdened care providers by systematically presenting summarized patient data, potentially enabling faster and more accurate decisions [43,52]. Such support is invaluable in high-pressure situations requiring rapid action based on comprehensive information. These findings are consistent with previous research that emphasizes the ability of chatbots to capture patient reports in a structured, comprehensive way [3,22]. Their conversational design facilitates higher engagement and satisfaction through interactive discussions [4,50]. This contributes to improved documentation of patient histories. Furthermore, automated information capture has been confirmed to increase both the efficiency and accessibility of health care by simplifying reporting processes [21,39].

While chatbots already promise success in supporting diagnostic processes, the required level of accuracy must be achieved for complex medical scenarios that require in-depth understanding and sound clinical judgment. The limitations of current systems are highlighted in the studies by Hennemann et al [45] and Jungmann et al [47], highlighting the need to improve the algorithms and decision-making processes to manage complex health conditions.

While the seamless integration of conversational agents into clinical workflows requires robust data infrastructures and user-friendly interfaces, such integration can drive adoption among care providers and patients if done in a secure manner [48]. Customized chatbots are required to serve different patient audiences and different facilities. Addressing these needs can increase patient engagement and satisfaction [48,50].

However, the development of such technologies requires careful consideration [56]. Rushing to release chatbots without thorough refinement and validation can lead to inaccuracies and potentially detrimental outcomes. These hastily deployed chatbots run the risk of failing to understand complex medical situations and recommending incorrect diagnoses or treatments. The use of chatbots requires caution and rigorous testing or validation to minimize the risks [57-59].

### Limitations

Although this systematic review provided useful insights, certain limitations must be acknowledged. As we only considered papers published in English, we may have overlooked important work published in other languages. In the future, a more comprehensive review that includes multilingual research could promote a more complete understanding of chatbots worldwide. The variability of study designs, patient groups, and health care contexts makes it difficult to draw definitive conclusions. Different studies, such as those by Denecke et al [39] and Faqar-Uz-Zaman et al [41], focused on different settings and patient groups, which influenced the results. Cross-sectional studies provide snapshots of usability, while RCTs provide robust evidence. Heterogeneity in demographics and health status also affects generalizability, as seen in the studies by Welch et al [53] and Wang et al [29]. Bias assessment frequently showed unmet STROBE criteria. Clear eligibility criteria and detailed methods could influence reliability. For example, Gashi et al [43] lacked defined selection criteria, and Jungmann et al [47] had a selection bias. Inconsistent reporting and lack of blinding in some RCTs, such as Wang et al [29], impaired internal validity.

The methodological quality of the included studies varied. At the same time, most observational studies demonstrated satisfactory quality, and a significant proportion fulfilled only some of the STROBE criteria. Additionally, the risk of bias assessment of the RCTs revealed a high risk of bias in one of the studies [41]. It is important to consider these limitations when interpreting the data and trying to understand how they relate to clinical practice. In addition, only published research has been included in this systematic review, which may lead to publication bias as studies with positive results are more likely to be published [41].

### Future Directions

Based on the findings and limitations of this systematic review, future research should focus on conducting more standardized and well-designed studies in this field. Emphasizing rigorous study designs, such as RCTs, with larger sample sizes and standardized outcome measures will enhance the scientific validity of the research and provide more substantial evidence of the effectiveness of chatbots in history-taking. Standardized outcome measures between studies are crucial for better comparability. Future studies should use measures such as diagnostic accuracy, patient satisfaction, engagement, and usability ratings. Instruments, such as the system usability scale or the technology acceptance model, could be used. Further investigation is needed to explore the specific contexts and patient populations where chatbots for history-taking may be most effective [29,50,53]. Different medical areas and health situations may present special considerations and challenges that could influence the implementation and acceptance of chatbot-based systems for taking medical histories, such as in the case of older people due to a more limited technical affinity or long medical histories in people with chronic illnesses.

Moreover, future research should address the challenges and limitations identified in this review. Efforts should be made to minimize bias and improve the methodological quality of studies. Conducting studies with more homogeneous patient populations and using consistent outcome measures would enhance the comparability and generalizability of the findings [39].

Finally, it would be valuable to explore the integration of chatbots with other technologies or interventions to optimize the history-taking process. The integration of chatbots with modern technologies, such as NLP, machine learning algorithms, and decision support systems, has the potential to significantly improve history-taking [21,46,51]. NLP could improve the ability to understand and interpret patient responses to the chatbot. The interactions will be more fluid and intuitive. Machine learning algorithms can be used to continuously improve chatbot responses based on patient interactions. This could lead to more accurate and personalized information. The integration of decision support systems can provide health care providers with real-time evidence-based recommendations. Research designs to investigate these integrations could include comparative studies for measuring differences in diagnostic accuracy, patient satisfaction, and efficiency between 2 groups. One group could use a simple chatbot, and another group could use an advanced chatbot with integrated NLP and machine learning.

### Conclusions

The systematic review provides an insightful overview of the use of chatbots in medical history–taking. The results show that chatbots can increase data completeness and user satisfaction. This can encourage patient engagement, and more accurate assessment can be achieved in a reduced timeframe. Chatbots can be used in primary care before the face-to-face visit. This would not only reduce the workload of medical staff but also enable more targeted interaction between patients and physicians. Future research should focus on different areas to improve the use of chatbots for medical history–taking. Larger studies and RCTs are essential for adequate validation. The use of chatbots needs to be investigated in different health care settings and with different patient groups, for example, in patients with chronic diseases, mental illness, or older patients and in people who are not tech-savvy. Another area that needs to be considered is analyzing the impact of chatbots on workflows in clinics or practices and the change in the doctor-patient relationship. In addition, data protection and security issues must be clarified to ensure the protection of patient data, especially considering the latest developments in AI models. These offer new opportunities for more precise and personalized interactions. Research should optimize these models for history-taking and integrate them into decision support systems for real-time evidence-based recommendations. If these areas are addressed, chatbots can significantly transform health care by improving efficiency, accuracy, and patient engagement, especially for underserved patient populations, as well as chronic disease management and real-time symptom assessment.

## Appendix

Multimedia Appendix 1 Search strategies conducted, overview of studies, quality assessment of included studies.

Multimedia Appendix 2 PRISMA Checklist.

## Abbreviations

AI: artificial intelligence
NLP: natural language processing
PICOS: participants, interventions, comparators, outcomes, and study design
PRISMA: Preferred Reporting Items for Systematic Reviews and Meta-Analyses
RCT: randomized controlled trial
STROBE: Strengthening the Reporting of Observational Studies in Epidemiology

## Data Availability

All data generated or analyzed during this study are included in this published article. All aggregate data collected for this review are available from the corresponding author upon reasonable request.
