# Supplementary material for: Transforming Health Care Through Chatbots for Medical History-Taking and Future Directions: Comprehensive Systematic Review
Source: JMIR Med Inform. 2024 Aug 29;12:e56628. doi: 10.2196/56628 (PMC11393511; doi:10.2196/56628)
Supplement: Multimedia Appendix 1 [file medinform_v12i1e56628_app1.pdf]

Table of Contents

Search strategies conducted *1*
Overview of studies.. *2*
Quality assessment of the included studies... *4*

Search strategies conducted

First search June 2023 (last search, updated July 2024)

Ovid MEDLINE(R) and Epub Ahead of Print, In-Process, In-Data-Review & Other Non-Indexed Citations, Daily and Versions, Last Date: 2/07/2024 12:19

#	Search Query
1	Chatbot*.ti,ab.
2	conversational agent*.ti,ab.
3	Chatterbot*.ti,ab.
4	chat-bot.ti,ab.
5	chatter bot*.ti,ab.
6	chat bot*.ti,ab.
7	conversational bot*.ti,ab.
8	Digital Medical Interview Assistant*.ti,ab.
9	1 or 2 or 3 or 4 or 5 or 6 or 7 or 8
10	anamnesis.ti,ab.
11	medical history.ti,ab.
12	history-taking.ti,ab.
13	anamnesis.ti,ab.
14	10 or 11 or 12 or 13
15	9 and 14

PubMed (Same use of keywords for IEEE/Open Science/Scopus)
Last Date: 3/07/2024 11:19
"Chatbot*" OR "conversational agent*" OR "chatterbot*" OR "virtual assistant" OR "intelligent virtual agent" OR "artificial intelligence chatbot" OR "AI chatbot OR "conversational AI" OR "dialogue system") AND ("anamnesis" OR "medical history" OR "history-taking" OR "medical interview" OR "patient interview" OR "medical questionnaire" OR "patient questionnaire"

Cochrane Central Register of Controlled Trials (CENTRAL)
Last Date: 3/07/2024 11:02

ID	Search	Hits
#1	("Chatbot*" OR "conversational agent*" OR "chatterbot*" OR "virtual assistant" OR "intelligent virtual agent" OR "AI chatbot" OR "conversational AI" OR "dialogue system")	420
#2	anamne*	1772
#3	history taking	10791
#4	#1 AND (#2 OR #3)	29

Overview of studies

1st Author, year	Title	Number of authors	Country	Year	Title of scientific journal	Topics and type of journal	Impact factor
(Denecke et al., 2018)	Digital Medical Interview Assistant for Radiology: Opportunities and Challenges	3	Switzerland	2022	dHealth 2022	Digital Health, Radiology, Medicine	N/A
(Denecke et al., 2022)	Self-Anamnesis with a Conversational User Interface: Concept and Usability Study.	4	Switzerland	2018	Methods of Information in Medicine	Methods in biomedical and health informatics	1.8
(Faqar–Uz–Zaman et al., 2022)	The Diagnostic Efficacy of an App-based Diagnostic Health Care Application in the Emergency Room: eRadaR-Trial. A prospective, Double-blinded, Observational Study	12	Germany	2022	Annals of Surgery	Surgery	13.79
(Frick et al., 2021)	Comparison of disclosure/concealment of medical information given to conversational agents or to physicians.	3	Germany	2021	Health Informatics Journal	Health Informatics	2.934
(Gashi et al., 2021)	Developing Intelligent Interviewers to Collect the Medical History: Lessons Learned and Guidelines.	5	Switzerland	2021	Studies in Health Technology and Informatics	Assistive Technology, Health information Technology	N/A
(Ghosh et al., 2018)	Quro: Facilitating User Symptom Check Using a Personalised Chatbot-Oriented Dialogue System.	3	Australia	2018	Studies in Health Technology and Informatics	Assistive Technology, Health Information Technology	2.717
(Heald et al., 2021)	Using chatbots to screen for heritable cancer syndromes in patients undergoing routine colonoscopy.	10	US	2020	Cancer genetics	Cancer	2.169
(Hennemann et al., 2022)	Diagnostic Performance of an App-Based Symptom Checker in Mental Disorders: Comparative Study in Psychotherapy Outpatients	4	Germany	2022	JMIR Mental Health	Mental Health	6.33
(Hong et al., 2022)	The AI Will See You Now: Feasibility and Acceptability of a Conversational AI Medical Interviewing System.	3	US	2022	JMIR Form Res	Technological innovations in healthcare and medicine	N/A

(Ireland et al., 2021)	Introducing Edna: A trainee chatbot designed to support communication about additional (secondary) genomic findings.	7	Australia	2020	Patient Education and Counseling	Counseling and communication in health care	3.467
(Jungmann et al., 2019)	Accuracy of a Chatbot (Ada) in the Diagnosis of Mental Disorders: Comparative Case Study With Lay and Expert Users	4	Germany	2022	Jmir Formative Research	Pilot studies and preliminary results	N/A
(Nazareth et al., 2021)	Hereditary Cancer Risk Using a Genetic Chatbot Before Routine Care Visits.	8	US	2021	Obstetrics & Gynecology	Gynecology and obstetrics	7.623
(Ni et al., 2017)	MANDY: Towards A Smart Primary Care Chatbot Application.	4	New Zealand	2017	Knowledge and Systems Sciences	Systems Sciences	N/A
(Ponathil et al., 2020)	Family health history collected by virtual conversational agents: An empirical study to investigate the efficacy of this approach.	5	US	2020	Genetic Counseling	Genetic Counseling	0.14
(Reis et al., 2020)	Addressing User Resistance Would Have Prevented a Healthcare AI Project Failure	5	Germany	2020	MIS Quarterly Executive	IT governance, technology	6.353
(Schneider et al., 2023)	Successful usage of a chatbot to standardize and automate history taking in Hymenoptera venom allergy	7	Germany	2023	Allergy	Allergy and clinical immunology	14.71
(Wang et al., 2015)	Acceptability and feasibility of a virtual counselor (VICKY) to collect family health histories.	8	US	2015	Genetics in Medicine	Genetics	8.864
(Welch et al., 2020)	Using a Chatbot to Assess Hereditary Cancer Risk.	6	US	2020	JCO Clinical Cancer Informatics	Cancer and informatics	4.52

Author	Title and abstract	Background/rationale	Objective	Study design	Setting	Participants	Variables	Data sources/measurement	Bias
(Denecke et al., 2018)	Conducted a cross-sectional study to develop a self-anamnesis application using rule-based AI, assessed via a usability test with 22 participants.	Challenge of motivating patients to provide comprehensive medical history during self-anamnesis.<br>Traditional methods (paper-based questionnaires) are less effective in gathering complete and accurate data.<br>A mobile application with a chatbot interface could encourage patients to provide comprehensive medical history during self-anamnesis.	Developing a self-anamnesis application with a chatbot interface to provide a better and high-quality information basis for initial consultation with therapists or physicians and to investigate the effects of therapy.	Experimental Design with an intervention to develop a self-anamnesis application using a chatbot interface.	Not mentioned	No eligibility criteria	Questions on stylistics, frequency, and favorite pieces for activities: listening to music, singing, playing an instrument, and moving/dancing. Some open-ended questions and others with a 4-level scale or multiple-choice answers.	Not mentioned	Not mentioned
(Denecke et al., 2022)	Carried out a cross-sectional study, utilizing a chatbot with a conversational user interface for digital medical history interview, demonstrating good usability.	Radiologists have limited personal interaction with patients.<br>Digital medical interview assistant (DMIA) with a conversational user interface (CUI) could improve patient satisfaction and diagnostic quality in radiology.	Illuminating medical history interviews from a radiologist's perspective and discussing their impact on diagnostic quality.	Observational Study analyzing conditions and requirements for medical history information in the radiological workflow.	Authors possess experiences with radiology workflow, reviewed existing data collection forms in radiology dept.	Authors with radiology department experiences; reviewed existing data collection forms from radiology departments	Concept for implementing a Digital Medical Interview Assistant (DMIA) into radiological workflow. A prototype DMIA was tested in a usability test with 5 subjects.	Not mentioned	Not mentioned
(Frick et al., 2021)	A cross-sectional study involving an online questionnaire showed that participants preferred disclosing medical information to a physician rather than a chatbot.	Patients often withhold medical information from physicians.<br>Using a chatbot-guided approach during the anamnesis process might encourage more forthright answering behavior.	Investigating how patients' attitudes toward disclosing and concealing medical information differ when interacting with conversational agents or physicians.	Not mentioned	German survey respondents divided into CA and physician disclosure groups.	212 participants recruited from acquaintances and organizations; 164 completed the survey and included in analysis	Self-disclosure index and self-concealment scale used to measure differences between CAs and physicians in the disclosure and concealment of medical information.	Not mentioned	Not mentioned
(Gashi F et al., 2021)	Cross-sectional study.<br>Implemented a rule-based chatbot as an intelligent interviewer, which asked up to 44 questions on medical history and patient's details.	Collecting accurate and complete medical information from patients is crucial for diagnosis.<br>A rule-based chatbot as an intelligent interviewer can facilitate this process.	With the experiences gained from this work, we developed an intelligent interviewer for another medical specialty, which is family medicine. In this paper, we aim to contribute the following: 1) We present the developed prototype called AnCha, including its concept, technical specialties, as well as benefits	Involves the development of a prototype called AnCha.	Not mentioned	No eligibility criteria	Not clearly defined.	Recommendations based on previous work on the chatbot Ana, which collects the music biography of a person.	Not mentioned
(Ghosh et al., 2018)	Case-control study.<br>Developed and evaluated a triage chatbot.<br>Chatbot engages in symptom discussions with patients.<br>Provides personalized condition synopsis.<br>Predicted user conditions correctly with 0.82 precision.	Many users seek medical information online before visiting a doctor.<br>A triage chatbot can help users identify health risks and seek appropriate medical care.	Describing the development process of Quro, a personalized healthcare assistant using natural language processing and machine learning, and evaluating its flexible and efficient basis for triage systems modeling.	Not mentioned	Not mentioned	No eligibility criteria	Not clearly defined.	Not mentioned	No efforts to address potential sources of bias.

(Heald et al., 2021)	Using chatbots to screen for heritable cancer syndromes in patients undergoing routine colonoscopy	Hereditary colorectal cancer syndromes are underdiagnosed.<br>An artificial intelligence-based chatbot could identify high-risk individuals and obtain consent for genetic testing.	Testing the utility of using a chatbot during colonoscopy to identify patients potentially at risk of hereditary cancer.	Not mentioned	Conducted at the Cleveland Clinic in Cleveland, Ohio, USA	English-speaking patients aged ≥18 years presenting for colonoscopy at the Cleveland Clinic	Subject demographics, progression through chat, responses to Colorectal Cancer Risk Assessment Tool (CCRAT), personal and family history, genetic test results, and communication with subjects.	Data collected through the chatbot, RedCap, and medical records	Limitations
(Hennemann et al., 2021)	Cross-sectional study.<br>Outpatient psychotherapy patients used Ada symptom checker app.<br>Accuracy was assessed based on agreement with therapist diagnoses.<br>Matched therapist diagnoses in 51% of cases for first suggestion, 69% within first 5 suggestions.<br>Accuracies for disorder	Accurate diagnosis of mental disorders is challenging.<br>An app-based symptom checker for mental disorders could aid in the diagnostic process.	Testing the diagnostic performance of a widely available symptom checker for mental disorders compared to therapists' diagnoses based on structured clinical interviews.	Observational Study with a comparative design to test the diagnostic performance of a symptom checker.	Conducted at psychotherapy outpatient clinic of the University of Mainz, Germany	Inclusion criteria: age ≥18 years, sufficient knowledge of German language; Exclusion criteria: acute suicidality, self-indicated acute mental or physical state	Diagnoses obtained from experienced therapists using structural clinical interviews.	Usability assessed using the System Usability Scale (SUS) and other items covering demographic and clinical characteristics, previous experience with ADA, and frequency of web-based health information seeking.	All text diagnoses were recoded
(Hong et al., 2022)	Cross-sectional study.<br>Participants tested an AI medical interviewing system.<br>Majority believed system can improve PCP's understanding of their health (80%).<br>70% agreed it can identify health risks to help maintain health.	Primary care providers face challenges in collecting detailed medical histories from patients.<br>A conversational AI medical interviewing system could improve data collection and patient engagement.	Evaluating the feasibility and acceptability of a conversational AI medical interviewing system among patients in primary care settings.	Not mentioned	Conducted at a family medicine clinic within a large academic medical center in Northern California	Participants aged ≥18 years and English-speaking	Web-based AI medical interviewing system to capture personal medical histories, family histories, and social determinants of health data.<br>Identify risk factors based on established guidelines for further evaluation.	Genie asked participants a series of questions to capture personal medical histories, family histories, and social determinants of health data.<br>Identify risk factors based on established guidelines for further evaluation.	Not mentioned
(Ireland et al., 2021)	Cross-sectional study.<br>Pre-test genetic counseling sessions analyzed.<br>Enhanced patient agency through considering personal and familial testing implications.<br>Health literacy improved by explaining genetic conditions and terms.	Providing relevant information for informed consent in genetic testing is crucial.<br>Using chatbots can support patients in making decisions about additional findings.	Developing a genomics chatbot, Edna, using transcript analysis of pre-test genetic counseling sessions for additional findings, complementing genetic counseling by providing genomic information.	Not mentioned	Not mentioned	Adults who had whole exome sequencing conducted for genetic condition diagnosis, received test results; 81 patients elected for additional findings analysis	Pre-test genetic counseling sessions audio-recorded and transcribed. De-identified transcripts analyzed for diverse set of consultations.	Data collected from face-to-face genetic counseling sessions, audio recordings, and patient notes made by genetic counselors after the consultation.	No efforts to address potential sources of bias.
(Jungmann et al., 2019)	Case-control study.<br>Evaluation of health app diagnostic quality for various mental disorders.<br>Moderate diagnostic agreement for adult mental disorders (kappa=0.64).<br>Low diagnostic agreement for childhood and adolescent mental disorders (kappa=0.40).	Digital health apps can aid in mental health diagnosis.<br>Many apps lack evaluation and evidence-based claims.	Addressing the need for research into apps for the screening or diagnosis of mental disorders and their importance in patient care.	Observational Study using a health app to diagnose case vignettes.	Not mentioned	No eligibility criteria	Ada—Your Health Guide app for screening and diagnostic support of health conditions. AI-based chatbot asks adaptive questions for medical or psychotherapeutic interviews, provides diagnosis and probabilities of possible and differential diagnoses.	Ada—Your Health Guide app used for screening and diagnostic support of health conditions, based on AI and constantly updated medical research findings.	Potential selection bias due to non-representative study participants or cases.<br>Performance bias if the app or diagnostic tool used was
(Nazareth et al., 2021)	Cross-sectional study.<br>Multicenter, retrospective study on chatbot use in cancer risk assessment.<br>64.2% of 95,166 patients engaged with the chatbot.<br>High completion rates: 89.4% for cancer risk assessment and 71.4% for genetic testing education.	Assessing an individual's risk for hereditary cancers is important.<br>Using a web-based chatbot can facilitate this process.	Examining user uptake and experience with a clinical chatbot automating hereditary cancer risk triage by collecting personal and family cancer history in routine women's health care settings.	Retrospective Observational Study analyzing the use of a clinical chatbot for hereditary cancer risk triage.	Conducted at 180 U.S. clinics across all four U.S. Census regions for preappointment assessment of cancer risk.	Patients with a valid email address or mobile phone number on file were invited	Chatbot with scripted conversation and natural language processing used to collect personal and family histories, and provide education on clinical genetics.	Not mentioned	Participants were stratified based on age groups using National Comprehensive Cancer Network guidelines. Stratification was also done

(Ni et al., 2017)	Cross-sectional study. Proof-of-concept for "Mandy", a primary care chatbot. Automates patient intake process with a mobile app. Includes analysis engine, symptom-to-cause mapper, and question generator. Combines data-driven NLP and knowledge-driven diagnostics. Evaluated using benchmark	Patients experience long wait times in primary care services. A mobile chatbot like Mandy can automate patient intake, reducing staff burden and improving patient experiences.	Not mentioned	Not mentioned	Not mentioned	No eligibility criteria	Not clearly defined.	Not mentioned	No efforts to address potential sources of bias.
(Ponathil et al., 2020)	Cross-sectional study. Evaluates the efficacy of a Virtual Conversational Agent (VCA) for collecting family health histories. Overall completion time longer for VCA, but task-specific times shorter for two out of five tasks. Lower overall workload observed with VCA. Significant improvement in user	Collecting family health histories is essential for early disease diagnosis. Using a Virtual Conversational Agent (VCA) can improve engagement and reduce overall workload.	Evaluating the efficacy of collecting family health histories through a virtual conversational agent (VCA) interface, a new method for gathering this information.	Cross-Over Study comparing the conversational approach with the standard interface tool for family health history collection.	Conducted at Clemson University's Human Systems Integration Lab	50 participants recruited with basic computer skills, aged ≥18 years, no previous experience with electronic FHx tools	Pre-test questionnaire assessed demographic data and Internet-related experience. Time taken to complete tasks for each tool and other measures analyzed.	Within-subject experimental design with the independent variable as the interface used. Pre-test questionnaire and printed fictional FHx scenario provided to standardize tasks.	Some actions, such as saving information multiple times, clicking on the help button, and typographical errors, were not considered errors in the
(Reis et al., 2020)	Case-control study. Discusses a failed AI project at a large hospital due to user resistance. Provides recommendations based on lessons learned for three types of AI: automation, decision support, and engagement.	User resistance to AI systems hinders their adoption in healthcare. Understanding user expectations and attitudes is essential for successful AI integration.	Implementing a cognitive agent and identifying the root causes of user resistance that led to the failure of the AI project at the hospital.	Not mentioned	Not mentioned	No eligibility criteria	No clealy defined.	Not mentioned	No efforts to address potential sources of bias.
(Welch et al., 2020)	Cross-sectional study. Reports on a campaign promoting the use of 'ItRunsInMyFamily' tool for hereditary cancers awareness. Data collected from across 3,783 U.S. counties, and a broad range of cancer types were reported.	Engaging adults in family cancer history (FHx) assessment is challenging. A user-friendly chatbot tool like ItRunsInMyFamily can increase accessibility and ease of use.	Assessing the characteristics of users and identifying opportunities to improve the family cancer history (FHx) collection tool.	Not mentioned	Data set analyzed by study team at the Medical University of South Carolina for 10,000 user campaign	No eligibility criteria	Data for each question included in the analysis, including data from users who didn't complete the assessment.	Not mentioned	Not mentioned

Author	Study size	Quantitative variables	Statistical variables	Participants	Descriptive data	Outcome data	Main results	Other analyses	Key results	Limitations	Interpretation	Generalisability	Funding	Total	Category rating
(Denecke et al., 2018)	Not explained	Some open-ended questions, others on a 4-level scale or multiple-choice answers	Not mentioned	Not mentioned	Age of the participants ranged between 19 and 73 years; the average age was 39 years.	22 participants (9 female, 13 male) completed the usability test and filled the correspon	Participants rated their technical competencies between 5 and 10, with an average of 7.77 on	None	Developed a system using a CUI for medical history collection. Patients felt motivated and at ease answering questions. Provides explanations and supports low health literacy patients.	Information extraction and mapping needed for free-text user input.	Not mentioned	Concept requires structured format for medical history data storage.	Not mentioned	12/22	B
(Denecke et al., 2022)	Not explained	Not mentioned	Not mentioned	Not mentioned	Not mentioned	AnCha chatbot with good usability, developed using RiveScript accessible through Telegram messenger	Not mentioned	None	Developed DMIA with conversational user interface using RiveScript. Good usability demonstrated in initial assessment.	Not mentioned	Not mentioned	Not mentioned	Not mentioned	9/22	C
(Frick et al., 2021)	164 completed the survey out of 212 participants	Statistical data were calculated for the Self-Disclosure Index and the Self-Concealment Scale (see Table 2). All variables were checked for normal distribution, and scales were assessed for	The survey was carried out with LimeSurvey, and statistical analyses were conducted using SPSS Statistics software (Version 25) and Jamovi	A total of 212 people participated in the study, of which 164 completed the survey (77.4%); 16 (9.7%) of the completed response sets were excluded	Participants of the physician group (M=2.66, SD=1.09) would rather disclose information than participants of the CA group	Physician group (M=2.66, SD=1.09) discloses more information than CA group (M=2.14, SD=1.12).	Chatbot group disclosed less information about drug use, sexual behavior, prescription drugs,	None	Patients prefer disclosing medical information to a physician. Disclosure to CA or physician is insufficient and incomplete. Willingness to disclose certain sensitive information is lower.	No direct interactions with a CA took place, potential bias in assessment.	Further research should develop a prototype of a CA for the anamnesis and validate it in real-life scenarios. Studies have shown that CAs are capable of assisting in the execution of work-related tasks and are able to accelerate internal processes. However, in the health-related context, CAs are not yet widely used, resulting in patients viewing them unfavorably. In addition, the	Further research needed for CA prototype validation and consideration of privacy and security aspects.	Open Access Publication Fund of the University of Duisburg-Essen	18/22	A
(Gashi F et al., 2021)	Not explained	Not mentioned	Not mentioned	Not mentioned	Not mentioned	AnCha chatbot collects medical history through 44 questions in about 10 minutes, sends data to	AnCha chatbot collects current symptoms, medical history through 44 questions, taking up	None	Not mentioned	Technical competency, patient willingness, and bond of trust with AnCha need assessment.	Not mentioned	Not mentioned	Not mentioned	8/22	C
(Ghosh et al., 2018)	Not explained	Not mentioned	Not mentioned	Not mentioned	Not mentioned	Initial evaluation of chatbot conducted with 30 clinical scenarios for triage urgency.	Chatbot achieved an accurate outcome in 83.3% for emergent care and 66.6% for GP/s self-	None	Chatbot achieved accurate outcomes in most cases. High recall of 100% for emergent care. Plan to test with more complex patient vignettes.	Limited consumer trust in chatbots due to design and AI limitations.	Chatbot achieved accurate outcomes in various cases, but limited consumer trust in chatbots remains. Continuous evaluation and engagement with users are necessary for improvement.	Limited consumer trust in chatbots; continuous evaluation and engagement with users necessary for improvement.	Not mentioned	13/22	C

(Heald et al., 2021)	Included a total of 487 subjects	The study collected quantitative data on time spent on study activities and subject interactions	Descriptive statistics, independent samples t-test, and Fisher's exact test were used for data analysis.	The target user groups are persons with an age of 16 and older, excluding children.	Descriptive data included subject demographics, time spent on study activities, and family history distribution.	Outcome data includes patient engagement, time spent per patient, and identification of	Successful engagement of patients with the chatbot. Found patients with germline	The study analyzed the response rate to the CCRAT questions and the utility of specific questions in the CCRAT.	Successful engagement of patients with the chatbot. Identified patients with germline pathogenic variant.	Lack of data on subjects' education or income, potential bias in patient responses.	Potential utility of using a chatbot in identifying patients at risk of hereditary cancer syndromes.	Not mentioned	Provided by Clear Genetics (now Invitae)	20/22	A
(Hennemann et al., 2021)	Not explained	Accuracy was calculated as the percentage of agreement along with the 95% CI for binomial distributions with the Agresti-Coull method.	The AC1 was calculated using AgreeStat version 2011.3 (Advanced Analytics). All other analyses were performed using SPSS (version 27; IBM Corp) and	Over the 1.5-year recruitment period, 159 persons were screened, 55 study participants remained after exclusions and early discontinuation.	On average, the participants were 33.41 (SD 12.79) years old, and 61% (30/49) were women.	22% used internet for health info, mean symptom duration=8.25 years, 39% reported past mental	ADA's condition suggestion aligned with therapists' diagnosis in 51% of cases, and was in the top	None	Approximately half of the cases had ADA's correct condition suggestion.	Thorough therapist diagnoses based on more information not available to the symptom checker.	Consider patients' and health professionals' views on the combination of traditional and digital diagnostic procedures.	ADA's diagnostic performance inconsistent between disorder categories and included low interrater reliabilities.	Not mentioned	19/22	A
(Hong et al., 2022)	Not explained	Patient-reported feasibility and acceptability ratings from the survey based on Likert-scale questions were used to measure performance expectancy, effort expectancy, and	Not mentioned	Twenty patients with a mean age of 50 years completed an interview with the AI system, including 12 females (60%) and 8 males (40%).	Participants who felt the system was easy to use tended to be younger.	Majority agreed that using AI medical interview system could improve understanding of health	Most participants agreed the system could help PCPs understand health (80%)	None	Majority of patients believed the tool could help PCPs understand their health.<br>Mixed results for effort expectancy and attitude toward the tool.	Small sample size, convenience sampling recruitment approach, limited generalizability.	Conversational agents could aid in capturing a more holistic view of patients and identifying disease risks earlier.	Feasibility and acceptability may be influenced by study-specific factors; replication in diverse settings needed for generalizability.	Not mentioned	16/22	B
(Ireland et al., 2021)	Not explained	All audio recordings were transcribed verbatim, but denaturalised, and a subset of consultations was selected for analysis of the de-identified pre-test transcripts.	Iterative analyses of the transcripts using NVivo Pro 11 (QSR International) was undertaken by CSIRO neuroscience and computer science	Not mentioned	Not mentioned	Chatbot Edna navigates patients through genetic info, customized questions, and	Not mentioned	None	Developed chatbot prototype "Edna" for genomic information.<br>Edna provides generic information but avoids personalized advice.<br>Uses logic reasoner and case-based reasoner for decision-making prompts.	Not mentioned	Edna complements genetic counseling, chatbots have unique contributions to genetic education. Further testing and modifications are needed.	Chatbots like Edna complement genetic counseling but cannot replace the ability to detect and respond to emotional cues. Further testing and modifications required.	Funded by State Government of Victoria and Melbourne Genomics Health Alliance	13/22	B
(Jungmann et al., 2019)	Not explained	The main outcome was the agreement between the main diagnosis of the case vignette in the textbook and the result given by the app, comparing diagnoses at the level of 4-digit codes in the ICD.	The main outcome was the agreement between the main diagnosis of the case vignette in the textbook and the result given by the app, comparing	Not mentioned	Descriptive data is shown in Table 1.	Agreement between app and textbook diagnosis: 68%, differential diagnoses agreement: 85%.	Percentage agreement with app diagnosis: 68% for adults, 42% for children/adolescents.	None	Health app "Ada" tested to detect mental disorders.<br>Moderate agreement for mental disorders in adulthood, low for childhood.	Small sample size, low ecological validity, performance influenced by individual values.	Health apps do not aim to replace doctors or psychotherapists, but provide low-cost support in the diagnosis of mental disorders.	Comparison of differences when patients do the input themselves needed; challenges in data security and standardization of quality assurance.	Not mentioned	16/22	B

(Nazareth et al., 2021)	Not explained	A satisfaction question was presented to users on completion of the chat, and the resulting values were averaged for an overall satisfaction score.	Analyses were conducted using statistical software R. A one-sided t test was used to compare various outcomes among different participant	A total of 95,166 individuals were invited to engage with the chatbot, and 61,070 (64.2%) patients engaged with the link.	Among the responders, most (96.3%) were female, and nearly half (45.5%) were 40–60 years of age.	1.9% adopted, <1% engaged in Spanish, nonusers were older (53.5 years vs 48.4 years, P<.001).	89.3% of users completed the risk-assessment section, 71.4% completed the educational section	None	27% triaged as high-risk for hereditary cancer syndromes, but few received testing. Hereditary cancer risk common in individuals receiving care or screenings.	Limited Spanish language option usage, lack of a non-invited comparison group.	Calls for routine genetic testing grow, ensuring access is not limited to the most privileged.	Findings may be generalizable to U.S.-based patients seeking routine care, but majority of patients were women.	Funded by Ambry, Invitae, and Progenity (industry support)	19/22	A
(Ni et al., 2017)	Not explained	Not mentioned	Not mentioned	Not mentioned	Not mentioned	Not mentioned	Not mentioned	None	Not mentioned	Not mentioned	Not mentioned	Not mentioned	Not mentioned	2/22	C
(Ponathil et al., 2020)	Recruited 50 participants, with 36 being the suggested size	Descriptive statistics were used to compare the time taken to complete the task, the number of errors made, and the results from various scales, and statistical tests were conducted for	Descriptive statistics were used to compare various outcomes between the VCA and the standard interfaces, and paired t tests	Not mentioned	Not mentioned	Participants were highly educated; 36% had four-year college degrees, 36% had master's degrees.	Participants took 53 seconds longer to complete tasks using the VCA interface, which slowed	Participants had unfavorable comments about the standard interface, except for editing information. Icons were	Compared efficacy of standard web forms and VCA for FHx information.<br>VCA interfaces performed significantly better in most measures.	Sample primarily well-educated, fictional data used, controlled setting, limited generalizability.	VCA approaches for collecting FHx data are easier to use and preferred by users over standard web interfaces.	Encourages the use of VCAs for collecting complex patient-entered data in health care; potential for new opportunities in health care.	Not mentioned	19/22	A
(Reis et al., 2020)	Not explained	Not mentioned	Not mentioned	Not mentioned	Descriptive data of the 16 interviewed physicians are provided.	Not mentioned	The case of a failed cognitive agent implementation in a hospital due to user resistance behaviors	None	Not mentioned	Not mentioned	Not mentioned	Not mentioned	Not mentioned	5/22	C
(Welch et al., 2020)	Not explained	Records with two or more invalid values were considered for removal because they were likely fake users.	Data were cleaned by removing impossible values, internally inconsistent values, and extreme outliers using collaborative	From November 1, 2019, to November 30, 2019, a total of 14,140 individuals accessed the ItRunsInMyFamily family cancer risk assessment URL.	Most female respondents were between the age of 40 and 59 years (92.5%; 4,636 of 5,014).	Respondents' average weight=193.7 pounds, average age at menarche =12.6 years,	Participants reported various cancer types in family history. Most commonly reported	None	54.4% of users proceeded beyond consent, 22.7% completed full assessment.	Difficulty identifying fake data, potential wording and algorithm problems.	Not mentioned	Study shows high engagement and interest in gathering FHx information using targeted marketing strategies.	Not mentioned	14/22	B

RCT studies

D1	Bias arising from the
D2	Bias due to deviations from intended interventions;
D3	Bias due to missing outcome
D4	Bias in measurement of the
D5	Bias in selection of the reported

Studies	D1		D2		D3		D4		D5
(Faqar-Uz-Zaman et al., 2022)	Low	Sample size: n=450 patients based on >30% inconsistent diagnoses and potential diagnostic efficacy improvement (70% to 85%) with Ada.<br>Statistical analysis: Categorical variables summarized as numbers (%), compared using χ2 test. Continuous variables summarized as median (range), compared using Mann Whitney U test/Kruskal-Wallis test.<br>Outcome evaluation: Spearman correlation	Low	450 patients analyzed (456 initially included, 6 excluded).<br>Study population divided into 3 subgroups: abdominal surgical, internal medicine, "other" departments.	Low	Total of 450 patients analyzed.<br>76% of patients followed up until day 90.	Low	Ada-App accuracy: 52.0% correct final diagnoses.<br>ER physician accuracy: 80.9% correct final diagnoses.<br>Combined assessment (Ada + physician): 87.3% efficacy rate.	Low
(Schneider et al., 2023)	Low	Fifteen patients each were randomized into a chatbot and a<br>control group. Chatbot group patients had completed the online<br>chatbot-based<br>anamnesis at home.Fifteen patients each were randomized into a chatbot and a control	Some concerns	Participants were aware of their assigned intervention during the trial	Low	All the outcome data was reported	Low	Method of measuring the outcome was appropriate	Low
(Wang et al., 2015)	Low	Patients recruited from Respect Registry at Boston Medical Center (BMC), serving underserved populations (73%).<br>Eligibility: 18+ years, English proficiency, current/former BMC patient.<br>Contacted via e-mail, letter, or phone; study RA confirmed eligibility and scheduled	Some concerns	No ITT analysis.	Some concerns	(>5% randomized not included)	High risk	Participants knew intervention and likely to influence outcome.	Some concerens
